# Shape-Memory Polymers in Dentistry: Systematic Review and Patent Landscape Report

**DOI:** 10.3390/ma12142216

**Published:** 2019-07-10

**Authors:** Alessandro Bruni, Francesca Giulia Serra, Andrea Deregibus, Tommaso Castroflorio

**Affiliations:** 1 Department of Surgical Sciences, CIR Dental School, Università degli Studi di Torino, Via Nizza, 230, 10126 Turin, Italy; 2 Department of Mechanical and Aerospatial Engineering (DIMEAS), Politecnico di Torino, C.so Duca degli Abruzzi, 24, 10129 Turin, Italy

**Keywords:** smart materials, stimuli responsive materials, shape memory polymers, dentistry, review, patent landscape report

## Abstract

Objective: To perform a systematic review (SR) of existing literature and a patent landscape report (PLR) regarding the potential applications of shape-memory polymers (SMPs) in dentistry. Search strategy: Clinical and Biomedical online databases (Pubmed, Medline via Embase, Scopus, LILACS, Web of Science, Cochrane Library), Materials Science and Engineering databases (IEEE Explore, Compendex, Proquest), Material Science and Chemical database (Reaxys) so as Patents databases (Questel-Orbit, Espacenet, Patentscope) were consulted as recently as January 2019 to identify all papers and patents potentially relevant to the review. The reference lists of all eligible studies were hand searched for additional published work. Results: After duplicate selection and extraction procedures, 6 relevant full-text articles from the initial 302 and 45 relevant patents from 497 were selected. A modified Consolidated Standards of Reporting Trials (CONSORT) checklist of 14 items for reporting pre-clinical in-vitro studies was used to rate the methodological quality of the selected papers. The overall quality was judged low. Conclusions: Despite the great potential and versatility of SMPs, it was not possible to draw evidence-based conclusions supporting their immediate employment in clinical dentistry. This was due to the weak design and a limited number of studies included within this review and reflects the fact that additional research is mandatory to determine whether or not the use of SMPs in dentistry could be effective. Nevertheless, the qualitative analysis of selected papers and patents indicate that SMPs are promising materials in dentistry because of their programmable physical properties. These findings suggest the importance of furtherly pursuing this line of research.

## 1. Introduction

Commonly, several dental materials (e.g., composites, cement, ceramics) were designed to survive for long periods in the oral cavity. They were designed in such a way that no interaction would occur between them and the oral environment. They were made to be passive and inert (or relatively inert), with minimal tissue response [1,2].

Interdisciplinary research is currently focusing on materials able to undergo purposeful change playing an active part in the way the structure or device works [1].

**Figure 1:**
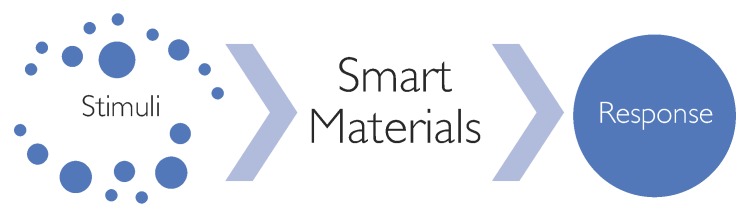
Schematic representation of smart materials’ behaviour.

Some of these materials, known as “smart” materials (SMs) or “(stimuli-)responsive” materials (SRMs), have a high capacity to sense and react according to environmental changes or external stimuli [3] (Figure 1): under a specific input, they produce a predictable and repeatable response or output. Such stimuli include: physically-dependent stimuli (e.g., temperature [4], electric fields [5], specific wavelength [6], ultrasound [7], magnetic fields [8], mechanical deformation [9]), chemically-dependent stimuli (e.g., pH [10], ionic strength [11], redox [12], solvent [13]), biologically-dependent stimuli (e.g., glucose [14], glutathione [15], enzymes [16], inflammatory metabolites [17]). A key feature of smart behaviour includes the ability to return to the original state after a stimulus has been removed [18].

The “smartness” feature in a material (or system) is determined by two differing mechanisms [19]:

Property change: specific input, resulting from the change in the conditions of the environment surrounding the material, affects the material’s molecular structure or microstructure causing a shift in one (or more) material’s properties (chemical, mechanical, electrical, magnetic or thermal). Changes are direct and reversible.

Energy exchange: specific input, resulting from the change in the condition of the environment surrounding the material, causes a shift in the energy state of material without affecting its properties. Changes are direct and reversible.

**Figure 2:**
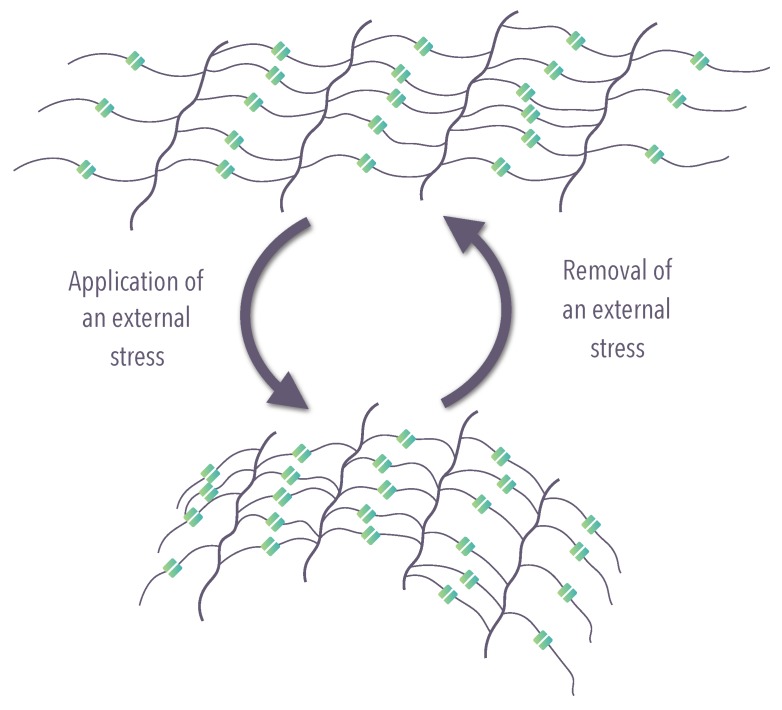
Schematic representation of the molecular mechanism of shape-changing material (SCM) (modified from Iqbal et al. [21]).

Among SRMs, one group is able to change its macroscopic shape in the presence of a proper stimulus [20]. According to their moving behaviour, it is possible to distinguish:

Shape-changing materials (SCMs): characterized by the shape-changing capability (SCC), which is defined as the materials’ ability to instantly change their shape gradually while they are exposed to a suitable stimulus [7]. They recover their original shape progressively as soon as the stimulus is terminated (Figure 2). SCC can be repeated several times.

**Figure 3:**
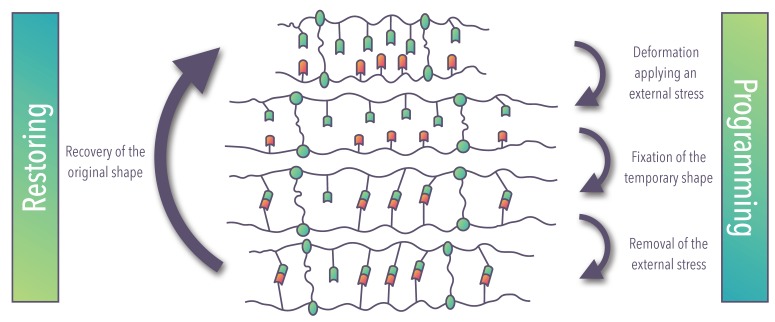
Schematic representation of the molecular mechanism of shape-memory material (SMM) (modified from Iqbal et al. [21]).

Shape-memory materials (SMMs): characterized by the shape-memory effect (SME), which is defined as the materials’ capacity to be deformed and fixed in temporary or dormant shape (programming), which remains stable until it is exposed to an appropriate stimulus (recovering) [22]. Once the original form is recovered the material can be programmed again (Figure 3); the SME is not an intrinsic material property, but a functionalization of material achieved combining a material’s molecular architecture along with a tailored processing and programming method [21].

In both cases, the basic molecular architecture is a suitable molecular network, but the mechanisms underlying the active movement differ [22]: SCC and SME differ in the degree of freedom defining the geometry of the movement as well as the reversibility of the action and the effect of the stimulus [23]. In SCM, the extent of shape recovery is sourced as a function of original molecular structure; on the other hand, in SMM, the extent of shape recovery is sourced as a function of fine programming leading to predefined temporary shape. The possibility to purposeful customize their moving behaviour, have increased the attractiveness of SMM over SCM.

**Figure 4:**
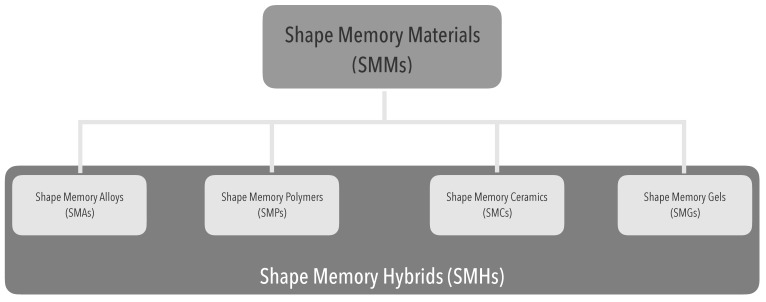
Shape-memory polymers within the shape-memory materials context.

The SME typify a distinctive feature of a multiplicity of materials [24] (Figure 4).

Shape-memory polymers (SMPs), also called actively moving polymers (AMPs), are a class of polymeric materials demonstrating SME: they can respond to several external stimuli such as temperature, magnetism, electricity, specific wavelength, moisture, pH and some specific chemicals [23].

Various types of polymers, such as polyacrylate copolymers [25], polynorbornene [26], segmented polyurethanes [27], segmented polyurethane ionomers [28], epoxy-based polymers [28], thiolene-based polymers [28], crosslinked polycyclooctene [29], crosslinked ethylene-vinyl acetate copolymer [30] and styrene-based polymers [31], and many more exhibit SME.

**Figure 5:**
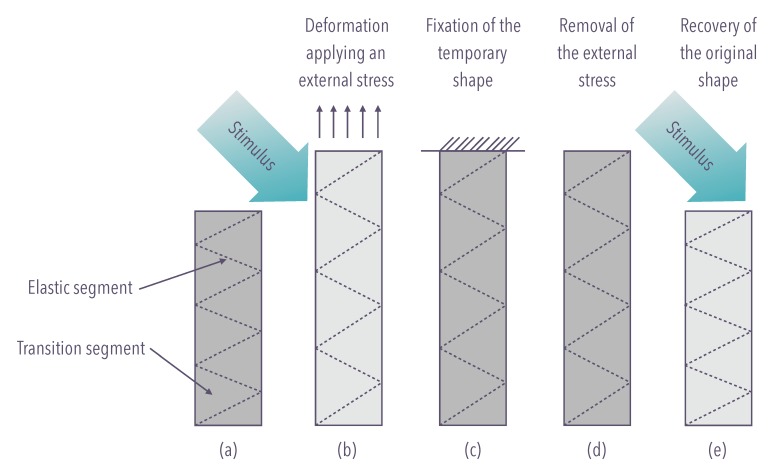
The mechanism for the shape-memory effect in shape-memory polymers is the dual-segment/domain system. (**a**) Native configuration; (**b**) external stimulus, inducing modulus drop, enables deformation after application of an external force; (**c**) fixation of the temporary configuration; (**d**) removal of the external force; (**e**) recovery of the native configuration.

The underlying mechanism for the SME in the shape-memory polymers is the dual-segment/domain system in which one is always hard/elastic maintaining dimensional stability, while the other can be soft/ductile or stiff depending on whether a right stimulus is presented. The former is called the elastic segment (or shape-fixing component), and the latter is the transition segment (or shape switching component) [32] (Figure 5).

The shape-memory effect has been appealing for its potential adoption in medicine since its discovery in metal alloys [33]. In dentistry, the introduction of nickel-titanium (NiTi) represented a recognized paradigm shift. In orthodontics, the use of NiTi allowed the employ of continuous and gentle force over a more extended time, fulfilling a multitude of clinical circumstances [34]. In endodontics, NiTi instruments facilitate the mechanical preparation of root canals with complex anatomy, reducing the incidence of canal aberrations [34]. Other applications of NiTi were reported in prosthodontics and oral surgery [34].

Compared with shape-memory alloys, SMPs exercise great attractiveness as a consequence of their significant elastic deformation ability, low cost (both for raw material and fabrication/processing), low density (which results in lightweight), ease of production and processing, tailorable physical properties, flexible programming, excellent chemical stability, biocompatibility and even biodegradability [24].

Because of these advantages, SMPs have great potential to penetrate virtually in any field of application such as smart products, transportation, biomedical devices and electronics. 

SMPs can be used widely in many areas such as biomedical devices, aerospace, textiles, energy, bionics engineering, electronic engineering, civil engineering, and household products [35]. Other applications might also be proposed, but we lack the space to discuss them all.

Surprisingly, this important area, in clinical practice as well as research, is relatively ignored, as evidenced by the scarcity of publications. The present work gives an overview of the available dental application of SMPs, with an appraisal of existing literature as well as technological monitoring based on invention patents, and highlights promising concepts and trends that may have the potential to promote the widespread use of this class of materials. It also attempts to address questions that would provide inspiration for future developments. 

## 2. Material and Methods

### 2.1. Protocol and Registration

This systematic review was reported in accordance with the guidelines of the Preferred Reporting Items for Systematic Reviews and Meta-Analyses (PRISMA) statement [36]. This comprehensive review protocol was registered into an online digital repository (figshare: https://doi.org/10.6084/m9.figshare.7977323.v1) and modified in April 2019.

### 2.2. Definition of the Research Question

The PO two elements format strategy [37] was used for framing research question (Table 1), that was formulated as follow: “*Do shape-memory polymers have potential applications in dentistry?*”, conforming to *FINER* criteria [38].

### 2.3. Eligibility Criteria

In Table 2 were shown the inclusion and exclusion criteria of this systematic review. No language restrictions were applied.

### 2.4. Information Sources, Search Strategy and Study Selection

A computerized database search was performed by two authors (BA and SFG) on January 21, 2019, to detect all peer-reviewed articles containing data regarding the use of shape-memory polymers in dentistry.

*Pubmed, Medline* (retrieved from *Embase*), *Scopus*, *LILACS*, *Web of Science*, *Cochrane Library*, *IEEE Explore*, *Compendex*, *Proquest*, *Reaxys* were the sources used to identify all significant studies regardless of the year, publication state or language. 

Documents not-indexed in available databases were directly hand-searched by the two authors.

The search strategy is described in Table 3.

Moreover, online patent databases (*Questel-Orbit*, *Espacenet*, *Patentscope*) were consulted to identify patent files related to the use of SMPs in dentistry.

Besides, patent searches were also conducted using International Patent Classification (IPC) with the code *A61C* (dentistry, oral and dental hygiene; class hierarchy in Table 4). That was because each patent may submit more than one IPC. The primary purpose of these codes was to narrow the search providing an effective tool to research and recover patents. The search strategy is described in Table 5.

The authors downloaded or manually entered references gathered from all the sources into a Reference Manager (Endnote X9, Clarivate Analytics, Philadelphia, PA, USA) in order to exclude studies and patents that were duplicated, apparently irrelevant or undeniably do not meet our inclusion criteria. For the review process, to complete a full analysis, the authors selected all studies (without blinding the names of the authors or publication dates) which appeared to meet the inclusion criteria or had insufficient data in the title and abstract to make a clear decision. The article selection process was carried out independently by two authors (BA and SFG). Full texts of the potentially eligible studies were retrieved and examined individually by two authors (BA and SFG) for compliance with the inclusion and exclusion criteria.

Articles which were not written in English were translated.

Potential disagreement, concerning the inclusion of studies, was solved through discussion and consensus with a third author (CT).

The search was completed with a review of references cited in the selected articles to identify additional studies not found in the initial search.

The selection of patents and the eligibility process were carried out along similar lines. 

## 3. Results

### Descriptive Analysis

**Figure 6:**
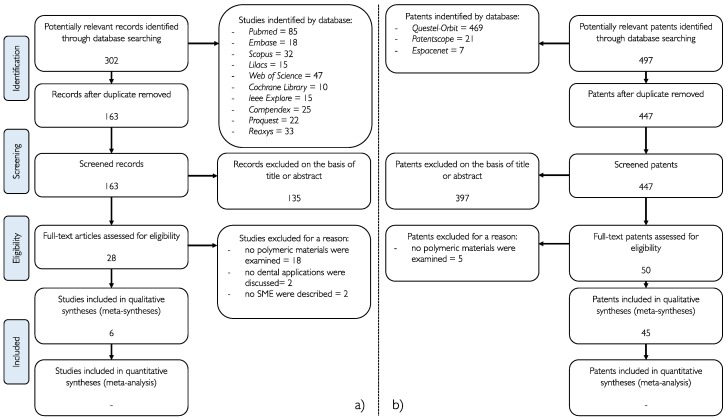
(**a**) Flow chart of the selection of the studies (performed according to the Preferred Reporting Items for Systematic Reviews and Meta-Analyses (PRISMA) [36] guidelines); (**b**) Flow chart of the selection of the patents.

The flowchart below (Figure 6) summarises the selection process for articles and patents.

Of the 302 articles initially recovered from all the databases screened (*Pubmed, Medline via Embase, Scopus, LILACS, Web of Science, Cochrane Library, IEEE Explore, Compendex, Proquest, Reaxys*), 135 articles were excluded because they were not correlated in any way to SMPs. 22 studies were excluded because they did not satisfy the inclusion criteria.

A total of 6 papers were included in the analysis. 

The modified Consolidated Standards of Reporting Trials (CONSORT) checklist was the methodological tool used by the authors to define the quality of all studies included in the systematic review [39]. The parameters considered were explained in Table 6. Each parameter was analysed in all articles and patents and accessed as reported (Yes) or not reported (No). The assessment was carried out separately by two reviewers (BA and SFG) and potential disagreements discussed with a third researcher (DA). The median (interquartile range (IQR)) quality assessment score of the 6 papers was 3.25 out of a maximum score of 15.

From the studies included, the following data were tabulated using predefined data extraction forms: title, author and year of publication, chemical composition, the field of application, type of study and main results (Table 7).

In the patent database (*Questel-Orbit, Espacenet, Patentscope*), the search strategy initially gathered 497 patents, with 397 being excluded after reading the title and abstract since they were not related to SMPs application in dentistry. Of the remaining 50 patents, 5 patents were excluded because they are related to inventions not entirely pertinent to dentistry. A total of 45 patents were included in the analysis.

From the patents included, the following data were tabulated using predefined data extraction forms: title, the publication number, the field of application and a brief overview of the inventions (Table 8). All the patents tabulated below provided the use of a SMPs as primary or auxiliary component in their embodiments.

The field of application with more patent registered was orthodontics (51%), endodontics (13%), prosthodontics (15%), oral surgery and implantology (15%).

**Figure 7:**
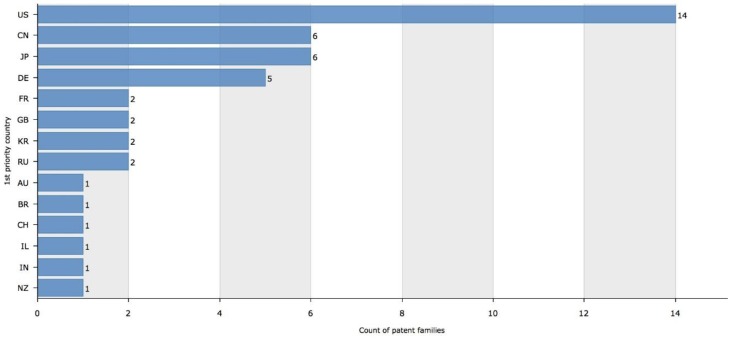
Patent families by priority country. Figure legend: US—United States; CN—China; JP—Japan; DE—Germany; FR—France; GB—United Kingdom; KR—Korea (South); RU—Russian Federation; AU—Australia; BR—Brazil; CH—Switzerland; IL—Israel; IN—India; NZ—New Zealand.

The countries with more patent applications were the United States (14 patents), Japan (6 patents), China (6 patents) and Germany (5 patents) (Figure 7).

## 4. Discussion

### 4.1. Overview and Limitations

In this study, both scientific publications and patents on shape-memory polymers were reviewed to describe the current state of the art of these promising materials in the field of dentistry. From the data available, the authors identified a progressive increase in studies and patents over the years, in accordance with the growing interest in this subject.

In this systematic literature review, six in-vitro studies, testing different types of SMPs, were selected: these studies showed heterogeneity in methods and the tested parameters. The analyzed papers showed an overall low quality. All of the studies failed in achieving the fundamental methodological domains: sample size determination, random sequence generation, allocation concealment, implementation details, blinding and publication of the full study protocol. Also, background and rationale, intervention description, outcome description, blinding, limitations, and funding resources were not always clearly reported.

On the other hand, technological monitoring consisted of 45 patents exhibiting the wide range of possible applications of these materials. 

In light of these findings, answering the research question was very challenging, and conclusions from the present systematic review should be interpreted with caution because there was not sufficient evidence supporting their transfer to clinical dentistry.

Nevertheless, some possible indications could be transferred to the dental community to improve the quality of future research on SMPs.

Among the analysed materials, scientific papers and patents identify some of the most promising SMPs. Undoubtedly, thermo-responsive shape-memory polymers are among the most commonly examined, having the ability to return from a deformed state to the original shape following a change in temperature. The mechanism for shape-memory behaviour, in thermal responsive SMPs, is the reversible activation and inactivation of polymeric chain motion in the switching segments respectively above and below the *transition temperature* (T_trans_) around which material changes from one state to another. T_trans_ could be either *melting temperature* (T_m_) or *glass transition temperature* (T_g_). Thermo-responsive shape-memory polymers have potential dental applications if these properties can be exploited at the temperatures of the oral environment; this has proven to be one of the main obstacles in the analysed works.

### 4.2. Orthodontics

The vast opportunities offered by using SMMs in orthodontics have not been revealed recently. In 1971 NiTi orthodontic archwires were introduced [90], representing the first medical application of an SMM. The ability to produce constant and lighter forces, reduced patient discomfort, less frequent readjustments have made them among the most common materials used for orthodontic devices (e.g., wires, coil springs, expanders, distractors) [34].

Consequently, shape-memory polymers could be a suitable alternative to shape-memory alloys. Their capability to deliver extrinsic mechanical stimulus that evokes a cellular response resulting in orthodontic tooth movement (OTM) was fully demonstrated [40,41,42,44]. Orthodontic archwires made by different SMPs (e.g., polyurethane-based [40], polyethyl methacrylate-based [41], and TPU-based [42]) were tested.

The mechanical and thermo-mechanical characteristics of the tested materials can be adjusted varying the cross-linking agent or hard segments content. Among the material’s proprieties, the modulus of elasticity has a great significance since a multiplicity of clinical settings requests a diversified range of wire flexibility. An adjustable modulus of elasticity, depending on treatment purposes, was feasible altering arbitrarily the chemical composition [40,41,43]. In the same way, selective control of the elasticity modulus in the various segments of the same wire might be possible (e.g., decreasing modulus in the areas of severe crowding; increasing modulus where more stiffness is desired) [41].

All the SMPs tested were triggered by a thermal stimulus; it was challenging to find materials able to recover their original shape, releasing light and stable force for an extended period, at the oral temperature. The intra-oral temperature fluctuations (e.g., consumption hot or cold substance [91]) make complex to predict accurately the extent of force expressed over time [44].

To overcome the lack of predictability, due to oral environment thermal instability, several alternative inputs (e.g., a particular wavelength [75]) were considered to drive SME.

According to an increasing demand for aesthetic treatments, the use of esthetic appliances has grown in popularity over recent years [92]. A significant feature of SMPs is the customization in colours (e.g., transparent, translucent, tooth-coloured), allowing an aesthetically pleasing appearance [40].

Similarly, clear aligners made by SMPs were introduced. The presented appliances, that meet the request for aesthetic procedures, need a lesser amount of replacements compared to traditional aligners, reducing treatment time and related costs [47,58,70,71]. Synthesizing polymers with more than two distinctive reversible phases [93] might be an additional challenge for the future.

Archwires and clear aligners were not the only application of SMPs explored in the orthodontic field. Elastic modules made by SMPs exhibited a lesser degree of force degradation at oral temperature for an extended period as their conventional counterpart [44]. However, the oral temperature modification induced by hot/cold liquid or food intake could significantly increase the recovery force degradation.

Moreover, an adjustable orthodontic band conforming to teeth of varied sizes was proposed [46]. The shape-memory properties allow the band to be adjusted between its first and second shapes for positioning on the tooth upon the application of a stimulus, enhancing the patients’ experience.

### 4.3. Endodontics

One of the aims of endodontic treatment is to achieve a three-dimensional, complete and hermetic sealing of the root canal system to prevent possible contamination by microorganisms or their products [94].

According to two articles by the same authors [4,43], the component ratio of *trans-1,4-polyisoprene* (TPI or pure gutta-percha) was refined to engineer an endodontic self-adjustable filling material able to seal firmly the whole endodontic space under a thermal stimulus. 

In the first article [43] the tested gutta-percha point adapted to the root canal, generating recovery stress that contributes to sealing the internal space of the root canal. The higher transition temperature of the material, compared with the intraoral temperature, represented the main shortcoming of the experiment. 

To overcome this limitation, the second article [4] reported a refinement in the material formula (regulating the cross-linking degree) that permitted obtaining a superior sealing ability under a thermal stimulus of approximately 37 °C. As shown previously, modifying the chemical structure of a SMP it is possible to customise also its physical proprieties to fit, potentially, any clinical circumstance.

Tailoring SMPs also predisposes them to implement additional features: an example was the radiopacity obtained incorporating bismuth oxychloride (BiOCl) pigments in the original polymer [77].

The adoption of SMP in the endodontic field was not limited to filling materials. 

Instrument fracture is among the most common undesirable events during endodontic therapy, delaying treatment completion and affecting the patient’s dental experience [95].

An endodontic instrument extractor tool for removing a fragment of a broken endodontic instrument that has become lodged within a root canal of a tooth, presented in a patent [59], might represent a congenial proposal to deal with this problem.

The active part of the endodontic instrument extractor comprises a hollow gripping body portion at the distal end made by shape-memory polymer in an expanded configuration with an inside cross-section initially greater than the dimension of the broken endodontic instrument. Upon heating, the shape-memory material returns to its unexpanded setting facilitating the removal of the broken endodontic instrument, gripping its proximal end.

### 4.4. Prosthodontics

SMPs are an exciting subject of investigation also in prosthodontics, restorative and oral surgery, where the lack of studies was compensated by the presence of several patents.

In prosthodontics, the presented applications aspire to optimise the daily practice through reducing chairside time and improving patient’s experience. Between various applications, provisional fixed prosthesis (with the invention of a temporary crown) and removable prosthesis (with a denture device) deserves special attention.

Provisional crowns are fundamental devices providing soft tissue health, proper occlusion and acceptable esthetic; however, they recurrently suffer from inadequate fitting [96]. 

To solve this problem, a patent [51] illustrated a pre-fabricated crown, moulded with shape-memory resin placed over the tooth abutment to create a provisional restoration. The peculiar feature of this device is the perfect adaptation and fitting with the abutment surface without cement. After deformation of the material, due to temperature changes, the provisional crown binds the abutment firmly, without further adaptation of the material.

Due to the gradual remodelling of alveolar bone in edentulous patients, complete dentures often require relining their internal surface to improve their stability and adaptation to the residual ridge. Relining can be done directly in the mouth or indirectly in a laboratory setting. Direct relining in the mouth is a fast procedure; however, potential oral mucosa irritation, bad odour, heat generation during the curing, and a weak bond between the reline material and denture base are areas of concern [97].

A denture engineered with a SMP lining the mucosa-side which repeatedly undergoes reversible changes could be an attractive alternative to direct relining. The mucosa-side part of the denture progressively adapts to the alveolar ridge, and the denture remains stable with minor patient discomfort and reduced need for periodic recalls.

The realization of a removable partial denture (RPD) including SMPs could be an additional captivating potential application. Indeed, RPDs remain an essential treatment option to edentulism compared with more costly alternatives. Traditionally, RPD frameworks were fabricated with metals (cobalt-chromium or titanium): although they were considered the materials of choice, their physical proprieties were not ideal. Therefore, the use of metal-free materials, including polymers, was investigated [98]. They undoubtedly present some advantages (e.g., better esthetic, cost-effectiveness, higher elasticity, easier reproducibility), nevertheless some disadvantages (e.g., faster deterioration than metal, possible cytotoxicity, minor mechanical strength) promote the need to improve materials for their fabrication [99]. SMPs could be an optimal choice because of their ease of milling and rheometric proprieties (which allow elastic deformation and recovery after the application and removal of stress), which should improve the distribution of mechanical stress associated with the function of RPDs.

### 4.5. Restorative

Restorative dentistry research is focused on the development of materials and techniques that mimic natural dentition [100]. As colour and aesthetics of teeth play a remarkable role in patient’s acceptance of restorations, selecting a correct shade to maximize the reproduction of natural teeth appearance become crucial for clinicians. Simplifying the selection of colour, reducing the number of shades, without compromising the esthetic outcome, leads to the necessity of new smart chromatic materials [101].

A composite material resulting from the combination of a shape-memory element and an optical change element material, which vary in response to an applied stimulus, was presented in a patent [76]. The possible employment of SMPs capable of optical shift and mimetism might be a breakthrough in the field of esthetic dental materials.

### 4.6. Oral Surgery/Implantology

Titanium and its alloys, thanks to their biocompatibility, mechanical proprieties and resistance to corrosion, were the materials of choice for dental implants. Despite their multiple advantages described in the literature [102], these materials lead to some issues (e.g., scattered radiation, occasional hypersensitivity, allergy, osteolysis and possible surface degradation). Although the numbers of studies were limited to achieve conclusions about dental utilisation, polymers (e.g., high-performance polymer polyetheretherketone) seem to possess favourable proprieties [98]. 

An implantable dental device, constituted by an artificial root, built including SMPs, for implantation into a cavity within the alveolar bone, was described in a patent [49]. The SMP can be activated from a deformed state to a relaxed state providing an expansion of the dental implant, resulting in a tight fit within the cavity. Advantageously, an instant fixation with a simple implantation procedure is obtained, and the osseointegration with alveolar bone could be rapidly achieved [103]. In a further refinement the collar portion of the device comprised resorbable polymer which may act as surface soft tissue growth promoter [49].

Another patent [87] introduced a device which not only enhances the opportunity of fixation into the bone but also reduces pain, discomfort and cost for both patient and dental surgeon. The invention provided a stent-like anchor (e.g., mesh, helix, tube) which is covered by porous materials formed into a sleeve. The choice of coverings with a specific porosity stimulates ingrowth of soft and hard tissue around the implant, thereby promoting healing and immobilization of the implanted device [103]. The porous covering could also provide the delivery of medicine or biologically active species for therapeutic or tissue ingrowth.

These patents are appealing, considering that the characterization of implant surfaces, to improve osteoblast adhesion or bacterial decontamination, is investigated by many authors [104,105]. 

Furthermore, according to a recent study SMPs were recently proposed as an active substrate for cells culture, supporting the feasibility of their use in tissue engineering targeted to scaffolds development [106].

### 4.7. Outlooks

Given the advances in computational sciences, digital imaging, innovative high-throughput sequencing and other molecular techniques (e.g., “omics” analyses including genomics, metabolomics, pharmacogenomics, transcriptomics) and improved understanding of oral biology, precision (or custom-made or tailor-made) dentistry may soon become a reality. 

As we progress towards precision dentistry, the material science is also progressing towards a fully customizable path. In this scenario, the rise of shape-memory polymers, with their high possibility of customization, seems to be the natural result of this demand. 

Furthermore, the use of three-dimensional manufacturing (3D printing) combined with shape-memory polymers could be another attractive domain to be developed for the near future. The change in the structural reconfiguration of 3D objects over time upon external stimuli has resulted in the emergence of novel ‘4D printing’ procedure [107].

The present systematic review and patent landscape report sharply focuses on the potentialities of these materials taking account of a low level of evidence supporting their application in a clinical setting. The main drawback was the almost exclusive use of thermal stimuli to activate shape-memory behaviour considering the thermic instability of the oral environment is. Further studies should focus on additional stimuli unbiased by surrounding conditions.

Whilst a significant increase in the number of pertinent publications has been made over the years, additional studies are required before effective therapies or suitable devices are implemented. A fair number of dental inventions made by SMPs were patented, but laboratory and clinical experiments would still be required before widespread use.

## 5. Conclusions

It was challenging to draw evidence-based conclusions that summarize the use of shape-memory polymers in dentistry owing to the diversity among studies and patents analysed, and the overall quality of the selected sources.

The results of this qualitative review and technological monitoring refer merely to in vitro studies and patents. The study aims to help to understand the mechanism behind the shape-memory polymers, despite further studies are necessary to corroborate these findings.

Taken together, current technology and results from the literature suggest that:

1. Polymers, have been tested successfully in vitro, starting to prove their worth; shape-memory polymers showed overlapping or better features towards existing materials (e.g., shape-memory alloys) even though such in-vivo comparisons have never been examined;
2. The chemical and structural diversity of available materials, while limited, has enabled the use of shape-memory polymers in a wide range of applications. A large number of shape-memory polymers have been developed and are currently being targeted for use in orthodontics.
3. The key feature why shape-memory polymers have been adopted is their ability to recover their original shape under selected stimuli; varying the chemical composition additional functions (e.g., biocompatibility, electric conductivity, stimuli-sensitive permeability, magnetic properties) could be potentially implemented. Targeted material design and synthesis could be tailored as appropriate.

Although the application of shape-memory polymers in dentistry is still limited due to the lack of a commercial supplier of dental materials, academic and inventors showed an increasing interest in testing the potential uses of this material family. There is much room for the improvement and further development of shape-memory polymers and consequently unfolds an exciting new field for materials selections in engineering design.

Unfortunately, aside from some preliminary works, the research field of SMPs is still nascent. Since research activities have strengthened over the last years, substantial advances can be expected soon.

## Figures and Tables

**Table 1:** Population/phenomena–outcomes (PO) framework for the research question.

**P (population/phenomena)**	shape-memory polymers
**O (outcomes)**	potential application in dentistry

**Table 2:** Study Selection Criteria.

Inclusion Criteria	Exclusion Criteria
Studies concerning the use of SMPs in dentistry	Review articles, editorials, letters, case reports, case series, thesis and dissertations
Patents related to dentistry (IPC: A61C7/00) and SMPs	Studies and patents not related to the dental application of SMPs

**Table 3:** Biomedical, Materials Science, Engineering and Chemical database search strategy.

Database	Search Queries	Results
Pubmed	((((shape-memory) or (shape memory)) and polymer*) or SMP*) and ((dental*) and (application*) or dentistry)	85
Medline via Embase	(1) shape-memory.mp. (2) shape memory.mp. (3) polymer$.mp. (4) SMP$.mp. (5) dental$.mp. (6) application$.mp. (7) dentistry.mp. (8) 1 or 2 (9) 3 and 8 (10) 4 or 9 (11) 5 and 6 (12) 7 or 11 (13) 10 and 12	18
Scopus	((((shape-memory) or (shape memory)) and polymer*) or SMP*) and ((dental*) and (application*) or dentistry)	32
Lilacs	((((shape-memory) or (shape memory)) and polymer$) or SMP$) and ((dental$) and (application$) or dentistry)	15
Web of science	((((shape-memory) or (shape memory)) and polymer*) or SMP*) and ((dental*) and (application*) or dentistry)	47
Cochrane Library	(1) shape-memory (2) shape memory (3) polymer* (4) SMP* (5) dental* (6) application* (7) dentistry (8) #1 or 2 (9) #3 and 8 (10) #4 or 9 (11) #5 and 6 (12) #7 or 11 (13) #10 and 12	10
Ieee explore	((((shape-memory) or (shape memory)) and polymer*) or SMP*) and ((dental*) and (application*) or dentistry)	15
Engineering village	(1) shape-memory.mp. (2) shape memory.mp. (3) polymer$.mp. (4) SMP$.mp. (5) dental$.mp. (6) application$.mp. (7) dentistry.mp. (8) 1 or 2 (9) 3 and 8 (10) 4 or 9 (11) 5 and 6 (12) 7 or 11 (13) 10 and 12	25
Proquest	((((shape-memory) or (shape memory)) and polymer*) or SMP*) and ((dental*) and (application*) or dentistry)	22
Reaxys	((((shape-memory) or (shape memory)) and polymer*) or SMP*) and ((dental*) and (application*) or dentistry)	33
		302

**Table 4:** International Patent Classification (IPC) class hierarchy.

**A**	Human Necessities
**61**	Medical or Veterinary Science; Hygiene
**C**	Dentistry; Apparatus or Methods for Oral or Dental Hygiene

**Table 5:** Patent database search strategy.

Database	Search Queries	Results
Questel-orbit	(((((shape-memory) or (shape memory)) and polymer+) or SMP+))/TI/AB/IW/CLMS/DESC/ODES/OBJ/TX and (A61C)/IPC	469
Espacenet	((((shape-memory) or (shape memory)) and polymer*) or SMP*), A61C	21
Patentscope	ALL:(((((shape-memory) or (shape memory)) and polymer*) or SMP*)) and IC_EX:A61C	7
		497

**Table 6:** Assessment of studies using the modified Consolidated Standards of Reporting Trials (CONSORT) checklist [39].

Ref.	1	2a	2b	3	4	5	6	7	8	9	10	11	12	13	14
Yung et al. [40]	NO	YES	NO	YES	NO	NO	NO	NO	NO	NO	NO	NO	NO	YES	NO
Masuda et al. [41]	NO	NO	NO	YES	YES	NO	NO	NO	NO	NO	NO	NO	NO	NO	NO
Kawaguchi et al. [42]	NO	YES	YES	YES	NO	NO	NO	NO	NO	NO	NO	YES	YES	NO	NO
Tsukada et al. [4]	NO	YES	NO	YES	YES	NO	NO	NO	NO	NO	NO	NO	NO	NO	NO
Tsukada et al. [43]	YES	YES	NO	YES	YES	NO	NO	NO	NO	NO	NO	NO	NO	YES	NO
Akihiko et al. [44]	NO	NO	NO	NO	NO	NO	NO	NO	NO	NO	NO	NO	NO	YES	NO

Information regarding the following parameters was judged as reported (Yes) or not reported (No): (1) Structured summary of trial design, methods, results, and conclusions; (2a) Scientific background and explanation of rationale; (2b) Specific objectives and/or hypotheses; (3) The intervention for each group, including how and when it was administered, with sufficient detail to enable replication; (4) Completely defined, pre-specified primary and secondary measures of outcome, including how and when they were assessed; (5) How sample size was determined; (6) Method used to generate the random allocation sequence; (7) Mechanism used to implement the random allocation sequence (for example, sequentially numbered containers), describing any steps taken to conceal the sequence until intervention was assigned; (8) Who generated the random allocation sequence, who enrolled teeth; (9) If done, who was blinded after assignment to intervention (for example, care providers, those assessing outcomes), and how and who assigned teeth to intervention; (10) Statistical methods used to compare groups for primary and secondary outcomes; (11) For each primary and secondary outcome, results for each group, and the estimated size of the effect and its precision (for example 95% confidence interval); (12) Trial limitations, addressing sources of potential bias, imprecision, and, if relevant, multiplicity of analyses; (13) Sources of funding and other support (for example suppliers of drugs), role of funders; (14) Where the full trial protocol can be accessed, if available.

**Table 7:** Articles included in the systematic review.

Article	Author (Year of Publication)	Chemical Composition	Application	Type of Study	Main Findings
Application of shape-memory polyurethane in orthodontic [40]	Yung et al. (2008)	polyurethane copolymer [4,4′-methylene bis(phenylisocyanate) + poly(e-caprolactone)diol (PCL) + 1,4-butanediol (4,4′methylene bis, polydiol, 1,4-butanediol)].	Orthodontics	in vitro	- polyurethane wire samples showed an average shape recovery of 80–85% at 30–50 hard segments wt.%; - Shape recovery (%) is directly proportional to hard segments wt.%; - the breaking stress (MPa) is inversely proportional to hard segments wt.%; - the elongation-at-break (%) is inversely proportional to hard segments wt.%; - the elastic modulus (MPa) is directly proportional to hard segments wt.%.
Development of an orthodontic elastic material using EMA-based resin combined with 1-butanol [41]	Masuda et al. (2011)	polyethyl methacrylate (PEMA-TA/HX resin) + 1-butanol	Orthodontics	in vitro	- the modulus of compressive elasticity (MPa), instantaneous modulus of elasticity (MPa), retarded elasticity (MPa), and viscosity (MPa·s) are inversely proportional to 1-butanol wt.%; - elastic (%) and permanent strain (%) are directly proportional to 1-butanol wt.%.
Effects of chitosan fiber addition on the properties of polyurethane with thermo-responsive shape memory [42]	Kawaguchi et al. (2016)	polyether-based thermoplastic polyurethane (TPU), TPU + biomass nanofiber (BiNFi-s), TPU + glass fiber	Orthodontics	in vitro	- elastic modulus (MPa) is directly proportional to wt.% BiNFi-s; - wt.% BiNFi-s did not influence the glass transition temperature.
Intraoral temperature triggered shape-memory effect and sealing capability of a transpolyisoprene-based polymer [4]	Tsukada et al. (2015)	trans-1,4-polyisoprene (TPI)cross-linked SMP-2 (Kuraray Corp, Kashima, Japan) + cis-1,4-polyisoprene (CPI) + Zinc Oxide + stearic acid + sulfur + dicumyl peroxide	Endodontics	in vitro	- shape recovery temperature (°C) and the recovery stress (MPa) are inversely proportional to CPI wt.%; - shape recovery temperature (°C) is inversely proportional to other cross-linking agents wt.%; - the shape recovery stress (MPa) is directly proportional to other cross-linking agents wt.%; - the relaxation modulus after 5s (MPa) is inversely proportional to CPI wt.% and directly proportional to other cross-linking agents wt.%; - the sealing at 37° is directly proportional to other cross-linking agents wt.%; - the shape recovery ratio (%) at 37° is directly proportional to other cross-linking agents wt.%.
Potential application of shape-memory plastic as elastic material in clinical orthodontics [44]	Akihiko et al. (1991)	polynorbornen	Orthodontics	in vitro	- polynorbornen samples showed a permanent deformation of 9.5% after 24 h and a constant and adequate recovery force (MPa) at ~50°, stretched at ~0.5mm/sec; - the recovery force (MPa) is directly proportional to % of stretching; - recovery force (MPa) is influenced by the environmental temperature (decrease with temperature over 43°; increase with temperature under 25°).
Temperature triggered shape-memory effect of transpolyisoprene-based polymer [43]	Tsukada et al. (2014)	cross-linked SMP-2 (Kuraray Corp, Kashima, Japan) + sulfur	Endodontics	in vitro	- shape recovery, recovery stress and relaxation modulus change as a function of temperature; - shape recovery (%) is directly proportional to temperature (°C); - the recovery stress (MPa) is directly proportional to heating (°C) and inversely proportional to cooling (°C); - the relaxation modulus after 5s (MPa) is inversely proportional to temperature (°C).

**Table 8:** Patents included in the patent landscape report.

Title	Publication Number	Field of Application	Invention Overview
Tridimensional dental aligner with activated pontic and activated bar alignment mechanics orthodontics using CAD/CAM [45]	600CHE2005	Orthodontics	A custom preprogrammed lingual bar to correct arch form comprising an active pontic with tooth moving potential.
Adjustable orthodontic band [46]	WO2003026526	Orthodontics	Orthodontic band with adjustable geometry to position and secure the band around a tooth.
Concealed orthodontic appliance [47]	CN204016523U	Orthodontics	Orthodontic appliance made of a transparent polymer material with biological safety to provide the orthodontic force required for moving teeth at the oral temperature.
Customized wire device for orthodontic alignment [48]	WO2014164779	Orthodontics	A customized wire designed to lock to bonded brackets rigidly.
Dental implant [49]	WO2008125852	Prosthodontics Implantology	An implantable dental device able to expand and provide a thigh fit into the alveolar bone.
Dental root canal filling material, method of filling root canal using the same, tubulus sealing type measuring device and method of tubulus sealing-type measurement [50]	JP2004135699	Endodontics	A self-expandable root canal filling material with shape recovery triggered by oral temperature.
Dental temporary coating crown and temporary securing method thereof [51]	JP2004337419	Prosthodontics	A temporary dental crown capable of tightly adapting to the abutment.
Dental tenon for fixing a tooth into a curved root canal comprises a core of long fibers embedded in a rigid matrix comprising a shape-memory polymer [52]	FR2863479	Endodontics Prostodontics	A dental post able to take place in a curved canal under the proper stimulus, capable of restoring its rigidity removing stimulus.
Dental wedge [53]	WO2015079424	Restorative	A dental wedge to separate, after activation, adjacent tooth and to secure a dental matrix against the tooth being restored.
Design configuration applied in a self-ligating bracket system [54]	WO2018022401	Orthodontics	A self-ligating bracket with a locking element that ensures a proper position of wires, preventing undesired displacements.
Device for atraumatic teeth extraction and fixer thereof [55]	RU0002470608	Oral Surgery	A device for atraumatic teeth extraction that applies a vertical displacement, similar to an orthodontic extrusion.
Device for fixing a prosthesis to a bone [56]	WO1994015544	Prosthodontics Implantology	A device with a component adapted for anchoring the device itself into the bone.
Device for the alleviation of snoring and sleep apnoea [57]	WO2009140720	Dental Sleep Medicine	An oral device to effect mandibular advancement for alleviating snoring and sleep apnoea.
Digitalized making method of dental orthodontic appliance and fixed appliance [58]	CN103405276	Orthodontics	A full digital method to produce a customized fixed appliance.
Endodontic instrument extractor tool manufactured from a shape-memory material and related kits and methods [59]	US7367804	Endodontics	An instrument extractor tool for removing a fragment of a broken endodontic instrument in a root canal.
Filling material pin for filling a tooth root canal is made from a flexible memory material which expands on heating to a specified temperature [60]	DE102005032005	Endodontics	Endodontic filling pin that expands on heating to a temperature of more than 30 °C.
Implantation device, implant and tool [61]	EP2291140	Prosthodontics Implantology	An implantation tool with a connecting structure able to retain the implant until a proper stimulus is applied.
Individuation orthodontic method based on shape-memory polymer arch wire [62]	CN103054651	Orthodontics	A full digital method to produce a customized archwire.
Integral fixed appliance [63]	CN203634309	Orthodontics	A manufacturing method to obtain an integrated orthodontic appliance.
It is just abnormal with munchkin soothing ring stick [64]	CN206534715	Orthodontics	A bite stick to provide additional orthodontic force under the action of mastication.
Method for producing a dental positioning appliance [65]	WO2014044720	Orthodontics	A method to produce a dental positioning appliance.
Method of tooth extraction (versions) [66]	RU0002491030	Oral Surgery	A device for atraumatic teeth extraction that applies a vertical displacement, similar to an orthodontic extrusion.
Mucosa-side material for denture, apparatus for manufacturing denture, and artificial tooth [67]	WO2002080806	Prosthodontics	A denture with a mucosa-side part that, after heating, can adapt itself to the shape of the alveolar ridge.
Multiple layered denture block and/or disk [68]	US20180055611	Prosthodontics	A multiple layered dental block for CAD/CAM milling.
Orthodontic appliance [69]	JP2005102953	Orthodontics	An orthodontic bracket able to correct its position and angle, without the necessity to repositioning on the tooth.
Orthodontic appliance by using a shape-memory polymer [70]	US20050003318	Orthodontics	A tray-type orthodontic appliance.
Orthodontic appliance having continuous shape memory [71]	WO2017079157	Orthodontics	A method to apply a continuous adjustment to an appliance.
Orthodontic brace with polymeric arch member [72]	US20080248442	Orthodontics	A removable arch connected to a series of brackets disposed on teeth.
Orthodontic bracket having wire fixing clip using shape-memory materials [73]	KR100691797	Orthodontics	A self-ligating bracket with an active wire fixation clip.
Orthodontic shape-memory band [74]	WO2017198640	Orthodontics	An adjustable orthodontic band conforming to teeth of different sizes.
Orthopedic jaw device, comprising bracket or buccal tube with cut-out to receive wire loop, at least partially formed from shape-memory plastics to allow easy fixing and replacement of the loop [75]	DE102004016317	Orthodontics	A bracket or buccal tube with a cut-out to receive a wire loop that under a stimulus converts to the original configuration causing retention of the wire loop in the cut-out.
Plural element composite materials, methods for making and using the same [76]	US20110140057	Miscellaneous	A method to produce a composite material resulting from the combination of optical shift and mimetism in response to an applied stimulus.
Radiopaque shape-memory polymers [77]	WO2010145741	Endodontics	A root-canal cone radiopaque comprising bismuth oxychloride (BiOCl) pigments as X-ray contrast agents.
Restorative dental appliances [78]	US20090246724	Orthodontics	A tray-type orthodontic appliance.
Self-adjusting orthodontic module [79]	US20090197216	Orthodontics	A self-adjusting orthodontic module that plays as force limiter in a fixed functional appliance (e.g., Herbst, Forsus).
Semi-thermoplastic molding composition having heat-stable custom shape memory [80]	WO1991012776	Miscellaneous	A preloaded impression tray.
Shape-memory material-based oral appliance production method and invisible appliance thereby [81]	CN104161596	Orthodontics	A method for manufacturing invisible appliance.
Shape-memory plastics articles and methods of processing same [82]	GB2340430	Endodontics	A root-canal cone capable of undergoing controlled radial expansion.
Shape-memory polymer orthodontic appliances, and methods of making and using the same [83]	WO2006071520	Orthodontics	A method of produce a component of fixed and removable orthodontic appliances.
Shape-memory resin, orthodontic appliance using same, and method for controlling viscoelastic property of shape-memory resin [84]	WO2012023454	Orthodontics	A method to produce appliances with controlled viscoelastic proprieties.
Shape-memory self-ligating orthodontic brackets [85]	WO2006014378	Orthodontics	A bracket with self-closing pair of opposing tie wings.
Silicone rubber composition and heat-shrinking cured product thereof [86]	JP6041435	Miscellaneous	A formula for producing a silicon rubber capable of heat shrinkage even at a temperature of ≤60 °C.
Surgical implant system for restoration and repair of body function [87]	US6299448	Oral surgery Implantology	A stent-like anchor which is covered by porous materials formed into a sleeve.
Temperature sensitive medical dental apparatus [88]	US5766004		A medical/dental apparatus which includes a portion connectable to part of a patient’s body.
Thermoplastic material and process for the production of a dental product [89]	WO2008064904	Miscellaneous	A thermoplastic device deformable at a temperature between body temperature and about 200 °C with at least one activator and/or receptor matched to an energy source for accelerating the heating process.

## Abbreviations

AMP	actively moving polymer
LILACS	literatura latino-americana e caribe a ciências da saúde
PLR	patent landscape report
PLR	patent landscape report
PU	polyurethane
SCC	shape changing capability
SCM	shape changing material
SM	smart material
SMC	shape-memory ceramic
SMc	shape-memory composite
SME	shape-memory effect
SMG	shape-memory gels
SMM	shape-memory material
SMP	shape-memory polymer
SR	systematic review
SRM	stimuli-responsive material
IEE	institute of electrical and electronics engineers
